# Effects of acute treatment with a tryptophan-rich protein hydrolysate on plasma amino acids, mood and emotional functioning in older women

**DOI:** 10.1007/s00213-014-3609-z

**Published:** 2014-05-25

**Authors:** E. L. Gibson, K. Vargas, E. Hogan, A. Holmes, P. J. Rogers, J. Wittwer, J. Kloek, R. Goralczyk, M. H. Mohajeri

**Affiliations:** 1 Department of Psychology, Whitelands College, University of Roehampton, Holybourne Avenue, London, SW15 4JD UK; 2 DSM Nutritional Products Ltd., Wurmisweg 576, 4303 Kaiseraugst, Switzerland; 3 School of Experimental Psychology, University of Bristol, 12a, Priory Road, Bristol, BS8 1TU UK; 4 DSM Food Specialties B.V., P.O. Box 1, 2600 Delft, The Netherlands

**Keywords:** Serotonin, Tryptophan, Emotion, Cognition, Depression, Memory, Wellbeing, Facial expressions, Functional food, Randomised controlled trial

## Abstract

**Rationale:**

Effective functioning of the neurotransmitter serotonin is important for optimal cognitive and emotional function. Dietary supplements able to increase availability to the brain of the precursor amino acid, tryptophan (TRP), and thereby enhance serotonin synthesis, can have measurable impact on these psychological processes.

**Objectives:**

This study involves a randomised controlled trial of a TRP-rich egg-white protein hydrolysate (DSM Nutritional Products Ltd., Switzerland) on plasma amino acids, cognition, mood and emotional processing in older women.

**Methods:**

Following a baseline test day without treatment, 60 healthy women aged 45–65 years received drinks containing either 2 or 4 g of TRP-rich protein hydrolysate product or 3.11 g casein hydrolysate as a control. One hour later, they undertook a 2-h battery of cognitive and emotional tests.

**Results:**

The TRP-rich protein hydrolysate produced the expected dose-dependent increase in the ratio of plasma TRP to competing large neutral amino acids. TRP-rich protein hydrolysate (2 g only) prevented both the decline in wellbeing and increase in fatigue seen over the test session in the control group. This treatment dose resulted in a significant shift in emotional processing towards positive words and reduced negative bias in assessing negative facial expressions. Effects on cognition were small and not statistically reliable and are not reported here. However, there was no evidence for any adverse effects.

**Conclusions:**

Consumption of a low dose of TRP-rich protein hydrolysate may have beneficial effects on emotional function that could promote feelings of wellbeing, possibly conferring resistance to deterioration in mood in healthy subjects or depressive episodes.

## Introduction

Recent work has established a key role for central serotonin (5-hydroxytryptamine (5-HT)) in the processing of emotionally relevant stimuli, as well as in cognitive function, especially memory, attention and information processing (Riedel et al. 2003). Thus, reduced 5-HT availability or turnover has been associated with impaired long-term memory but improved focused attention (Booij et al. 2005; Sobczak et al. 2002b); by contrast, increased 5-HT release, for example, after loading with high doses of the precursor amino acid l-tryptophan (TRP) can result in reduced vigilance (Schmitt et al. 2006; Silber and Schmitt 2010).

These effects of reduced 5-HT function are thought to underlie changes in emotional processing and cognitions associated with depression, anxiety and poor stress coping (Robinson and Sahakian 2009); thus, antidepressants may exert their beneficial effects by normalising deficits in central 5-HT transmission, although the precise mechanism for this remains elusive (Cowen 2008).

The strongest evidence for a role for functional 5-HT deficits in depression arises from studies using amino acid loads devoid of the precursor amino acid TRP to suppress 5-HT synthesis (acute TRP depletion (ATD)) (Young 2013; Young et al. 1985). This results in a substantial (e.g. >70 %) drop in plasma TRP within 4–6 h, and can induce dysphoric mood, especially in recovered depressives or those genetically at risk (Silber and Schmitt 2010). Essentially, reducing TRP access to the brain in this way tends to mimic the cognitive biases seen in depressed populations, such as impaired memory for, or attention to, positive vs. negative information (Mathews and MacLeod 2005). Thus, recognition of emotions in faces can be affected by ATD: for example, recognition of fear was reduced in women (Harmer et al. 2003). Furthermore, using asymmetry of the frontal electroencephalographic (EEG) power spectrum, an indicator of risk of depression and poor psychological wellbeing, Allen et al. (2009) showed that the extent to which ATD altered the EEG asymmetry in at-risk and healthy subjects predicted occurrence of depression in the following 6–12 months.

Conversely, an important question is whether similar behaviours can be improved by increasing/facilitating entry of TRP into the brain and thus elevating 5-HT synthesis. Indeed, there has been long-standing clinical and research interest on effects of oral or intravenous dosing with TRP (Fernstrom 2012). One particularly relevant finding has been the induction of a positive bias in processing of emotional faces in women (Murphy et al. 2006). However, a complication of oral TRP as the free amino acid is that it increases release of several hormones including growth hormone, cortisol and prolactin (the latter thought to indicate increased central serotonin—and dopamine—activity) (Porter et al. 2003).

It has long been recognised that serotonin synthesis in the brain can be affected by dietary manipulations that change availability of TRP to the brain. The rationale for interest in dietary manipulation of serotonin arises from the following facts: (i) TRP is an essential amino acid that has to be obtained from the diet and cannot itself be synthesised in the body; (ii) in the synthesis of 5-HT, the rate-limiting enzyme step involves conversion of TRP to 5-hydroxytryptophan (5-HTP) by TRP hydroxylase, prior to synthesis of serotonin; and (iii) TRP hydroxylase is not fully saturated by substrate, and so synthesis is sensitive to substrate availability, i.e. the brain level of TRP. Thus, increasing or decreasing TRP ingestion could in principle raise or lower 5-HT synthesis. However, the situation is more complex because of a specific transport system through the blood brain barrier (BBB) and liver metabolism. To enter the brain across the BBB, TRP has to compete for uptake not only against other amino acids, in particular a group known as the large neutral amino acids (LNAA), especially the branched chain amino acids, leucine, isoleucine and valine, but also phenylalanine and tyrosine (the precursors for catecholamine transmitter synthesis). For this reason, the ratio of plasma or serum-free TRP/LNAA is typically thought to be the best predictor of uptake of TRP into the brain. TRP is the scarcest amino acid in most dietary protein, so eating a protein-rich meal normally reduces this ratio: conversely, eating a high-carbohydrate, low-protein meal will raise the ratio, particularly as the rise in insulin leads to peripheral tissue uptake of the competing LNAA (Fernstrom and Fernstrom 1995).

This mechanism has been suggested to underlie dietary effects on mood and performance, such as calming after high-carbohydrate meals vs. arousal after protein-rich meals (Gibson and Green 2002; Hoyland et al. 2008). More specifically, there is evidence that individuals with inherently insufficient 5-HT activity, who may be prone to neuroticism or depression, may be most likely to benefit, in mood and cognition, from a 5-HT-enhancing high-carbohydrate, low-protein diet (Markus et al. 1998, 1999).

In recent years, a method has been developed to enhance TRP availability to the brain, and so potentially 5-HT function, by administering TRP-rich dietary proteins: the most published example is the whey protein α-lactalbumin. The effects of this protein are usually compared with responses after another protein, typically casein hydrolysate (another milk protein), which has lower levels of TRP but greater amounts of the competing LNAA. In addition, the proteins have typically (though not always) been given on two occasions, as drink supplements to high-carbohydrate meals (Schmitt et al. 2005).

α-Lactalbumin has been shown to enhance (or correct) serotonin function (indexed by prolactin release) and cognition, and to reduce cortisol release, in stress-prone (more anxious) participants (Markus et al. 2000, 2002). Another ‘vulnerable’ group investigated is women suffering from premenstrual syndrome, in whom memory may be impaired premenstrually. α-Lactalbumin attenuated deficits in delayed memory for abstract patterns in this group (Schmitt et al. 2005) , and in recovered depressives and healthy subjects (Booij et al. 2006). There are also reports of increased perception of emotional faces with TRP-rich protein in women (Attenburrow et al. 2003): however, so far, effects on emotional face processing appear to be weaker or less specific than dosing with TRP alone (Scrutton et al. 2007).

LumiVida™, a product developed by DSM Nutritional Products Ltd., is based on a similar premise, but is an egg-white protein hydrolysate formulation that contains fewer competing LNAAs. LumiVida™ has recently been shown to be more effective in raising plasma TRP/LNAA ratios than either α-lactalbumin or TRP alone (Markus et al. 2008; Mitchell et al. 2011). There is preliminary evidence that this high (12 g) dose of LumiVida™ may be effective in improving mood (Markus et al. 2008) and in enhancing psychomotor and vigilance performance, at least in individuals more resilient to stress (Markus et al. 2010).

This report concerns the acute effects of two relatively low doses of LumiVida™ on emotional and cognitive function in healthy middle-aged women. In selecting the doses, the intention was to detect the smallest efficacious doses for modification of plasma TRP/LNAA ratio and behaviour. Women were chosen because (a) they are more prone to suffer from anxiety and depression over their lifetime than men and (b) there is evidence that women may be more sensitive to behavioural effects of 5-HT manipulations (Murphy et al. 2006). Older women were also more likely to have relatively stable sex hormone levels, thus avoiding another potential confound (Schmitt et al. 2005). The timing of testing (1 to 3 h after the treatment drink) was intended to follow peak increases in plasma TRP/LNAA ratio (Collins et al. 2013; Fernstrom and Fernstrom 1995), with the expectation of maximising any impact of early increases in 5-HT synthesis and release on outcome measures.

Based on the findings described above, it was hypothesised that (a) acute treatment with LumiVida™ would cause dose-dependent increases in plasma TRP levels and TRP/LNAA ratio; (b) LumiVida™ would improve mood, psychological wellbeing and emotional processing, in line with effects seen with enhancement of serotonin function, including reducing negative bias in face recognition and perception of emotional stimuli; and (c) LumiVida™ would improve performance, in a dose-dependent manner, on tests of hand-eye coordination, verbal memory, working memory, sustained attention and driver hazard perception. Importantly, this range of tests also allows the study to determine whether these treatments produced any negative effects, such as reductions in cognitive function or sedation.

## Methods

### Design

This study was double-blind, randomised and controlled with treatment between subjects. Sixty women were randomised to control (casein hydrolysate, 3.11 g), LumiVida™ (egg-white protein hydrolysate, 2 g) or LumiVida™ (4 g). Prior to any testing, randomisation was stratified within three age subgroups (using Excel) to ensure an even spread of ages across treatment groups. Three participants failed to begin the study after randomisation (personal reasons). The final sample sizes were: control (*N* = 19; 2 g (*N* = 20); 4 g (*N* = 21). Each participant received either a single dose of the egg-white protein hydrolysate supplement, or casein hydrolysate as a protein control, on the treatment test day. The sample sizes were chosen to be towards the larger end of the range used in previously published studies of effects of TRP-rich proteins on amino acids, cognition and/or emotion, i.e. from *N* = 14 to *N* = 23 per group (Markus et al. 2008, 2010; Merens et al. 2005; Scrutton et al. 2007). Cognitive and emotional function, mood and physical sensations were assessed at baseline (screening day) and between 60 and 180 min after supplement consumption on the test day. The between-subjects design reduces practice effects as well as order effects that may result from differential tolerance, etc., when using within-subject designs in multiple dose treatment studies.

### Treatment

#### Active condition

LumiVida™ (DSM Nutritional Products Ltd., Kaiseraugst, Switzerland) is a hydrolysed, enzymatic digest of a dietary egg-white protein manufactured by using a proprietary mix of enzymes from a dietary source to provide a high-quality source of peptides. LumiVida™ contains a guaranteed minimum quantity of bioactive TRP-containing peptides. The molar ratio of these TRP peptides to peptides containing the LNAAs valine, isoleucine, leucine, tyrosine and phenylalanine is approximately 0.2. LumiVida™ is intended for use in food products such as beverages, bars, yoghurt drinks, etc., and these doses were considered (at the time) to be (a) sufficiently active on TRP entry to the brain (b) at a commercially viable level. In this study, LumiVida™ was taken as a citrus-flavoured non-nutritively (Acesulfame) sweetened beverage; 2 and 4 g of LumiVida™ were used, which contain approximately 0.13 and 0.27 g TRP, respectively.

#### Placebo condition

Casein hydrolysate (3.11 g; primary milk protein; DSM Nutritional Products Ltd., Kaiseraugst, Switzerland) was used to provide an intermediate amount of energy and total protein, in the placebo beverage, relative to the active treatments. Casein hydrolysate is low in TRP and so will not raise serotonin synthesis.

Both treatments were supplied in sachets of powder, administered as suspensions in approximately 150-ml tap water. Double-blind randomisation was carried out by means of a label code associated with a specific participant ID number, whose meaning was known only to the supplier. Participants were allocated randomly to these numbers.

### Participants

Participants were 60 women, aged 45 to 65, physically and mentally healthy, as defined by a brief medical history check (not concurrently receiving medical/pharmacological treatment except mild painkillers), free of gastrointestinal complaints, diabetes and cardiovascular disease, not in pain and not diagnosed with a psychiatric disorder (no treatment for depression or anxiety and other psychiatric disorders). Body mass index was required to be above 18 and below 35.

In addition, it was necessary to establish menstrual status prior to arranging the first (baseline) visit, as any premenopausal participants needed to be tested in the follicular phase (within 2 weeks of the start of menstrual bleeding). Menstrual status was established by questionnaire once verbal consent was established.

Recruitment was via notices in local newspaper/magazines, women’s organisations, campus posters, e-mail announcements and word of mouth (not family members). A participant information sheet was sent or read out to participants initially expressing interest, and if this seemed acceptable (verbal consent), they were invited for a screening interview and baseline test morning. The following restrictions applied: (i) No alcohol on the day before testing (including baseline day), (ii) usual caffeinated drink (if taken) to be drunk before 0800 hours on each test day and (iii) no other food or drink except water to be taken from 2200 hours the previous evening, until after testing has ended.

The study was approved by the University Ethics Committee of the University of Roehampton, in accordance with the 1964 Declaration of Helsinki. All participants gave informed signed consent before participating in the study.

### Procedure

Prior to the baseline test, participants were sent four brief questionnaires to assess personality characteristics that have been associated with serotonin function: Dutch Personality Inventory—Neuroticism scale (21 items), translated into English by a native Dutch speaker and refined by a native English speaker (Luteijn et al. 1975); this questionnaire has been used in previous studies of dietary manipulation of serotonin (Markus et al. 1998, 2010); Depression Anxiety and Stress Scale (DASS; 21 items, 1-week retrospective; Antony et al. 1998); Aggression Questionnaire—short form (Bryant and Smith 2001); Barratt Impulsiveness Scale—15-item short form (Spinella 2007).

Schedule of testing is given in Table 1: participants arrived at the laboratory between 0830 and 0850 hours. At the screening interview, the information about the study was repeated, and they were asked to read and sign the consent form, a copy of which they kept. Then, the brief health history and demographic information was checked, which was followed by measurements of their height and weight. Next, the participant was offered water to drink, and then asked to complete a series of 28 mental and physical sensation ratings on a computer screen. This computer-based mental and physical sensations scale (MAPSS) was run on E-Prime (v.2, Psychology Software Tools, Inc., PA, USA), questions were randomly sequenced and the scale ranged from 1 = 'Not at all' to 9 = 'Extremely'. This test was repeated once again at the end of all testing. MAPSS has been found to be sensitive to mood changes following psycho-pharmacological manipulations (Rogers et al. 2010).

**Table 1:** Cognitive test battery schedule

Time	Task
09:20	Mental and physical sensations scale (MAPSS)
09:25	National adult reading test (or drink on treatment test day)
09:30	Rest 1 h
10:30	Simple reaction time
10:40	Rotary pursuit task
10:55	Rest (or second blood sample of Day 2)
11:10	Verbal recognition memory (VRM); immediate recall/recognition
11:15	Match to sample (MTS): colour patterns for visual matching, attention, motor skill and reaction times
11:20	Rest
11:35	VRM: free delayed recall: paper test
11:40	Rapid visual processing (RVP): number sequence pattern detection
11:50	VRM: delayed recognition
11:55	Affective go/no-go (AGN): positive/negative/neutral target words
12:15	Emotional facial expression task: perception of angry, fearful, sad, surprised, disgusted and happy expressions
12:25	Rest
12:30	Driving hazard perception task
12:50	MAPSS

Participants then completed the national adult reading test (NART; 2nd edition, Windsor NFER-Nelson, 1991), which provides a measure of stable verbal IQ and was only given on the baseline test day. This involves participants reading aloud a series of 50 words, presented one by one on cards: they were scored for number of errors in pronunciation. After a rest of approximately 1 h, the baseline set of cognitive tests started at 1030 hours, in line with the subsequent test day, and lasted 2 h and 20 min including breaks.

### Treatment test session

As before, participants arrived in the laboratory for 0830–0850 hours and were asked about their current health, in case any adverse events needed to be noted, and also whether they experienced any stress so far that morning. The test schedule followed that of the baseline day, except that (i) two blood samples were drawn during the morning using a butterfly needle inserted in a vein in the antecubital fossa of the forearm and (ii) the freshly prepared experimental drink was given immediately after the first MAPSS, followed by a 1-h rest before cognitive testing began. Just prior to the first test (reaction time), a buccal (cheek) cell swab was taken, for later DNA assay (data not presented here). The first blood sample in the morning was drawn in a seated position prior to the initial MAPSS ratings, while the second sample was drawn 90 min later at the time of expected peak changes in plasma TRP and TRP/LNAA levels (Mitchell et al. 2011), following completion of the rotary pursuit task but before starting the critical memory tests. This blood sample was taken in order to verify that the LumiVida™ treatment raised the plasma TRP/LNAA amino acid ratio in the expected dose-dependent manner, compared with the control. The blood (6 ml) was collected in a Vacutainer™ lithium-heparin tube, shaken, stored on ice and processed within 2 h in the Clinical Laboratory at the University of Roehampton (see ‘Blood assays’ below). After cognitive testing ended, participants completed a ‘Tolerability’ questionnaire to assess any symptoms they may have experienced and were asked whether they believed they had been given a placebo or active treatment drink. Then, participants were debriefed, given a £60 store voucher card, reimbursed travel expenses up to £10 and offered free lunch in the university canteen.

### Test battery details

Considering space restrictions, detailed test information is only given for those tests that produced significant results. Performance on the following cognitive tests did not show statistically reliable treatment effects: simple and sustained reaction time (Rogers et al. 2010), rotary pursuit task (model 30014A, Lafayette Instruments), verbal recognition memory, match to sample visual search, rapid visual information processing (the latter three are from CANTABeclipse v3, Cambridge Cognition Ltd.; http://www.camcog.com/cantab-tests.asp) and the driver hazard perception test (from the driving theory test, professional version 2008–2009, Oasis Software Ltd.). Further information about these tasks is available from the manufacturers and on request from the authors.

#### Affective go/no-go task (AGN; CANTABeclipse v3)

This test assesses information processing biases for positive and negative stimuli (Cambridge Cognition Ltd.; http://www.camcog.com/cantab-tests.asp). The test consists of several trial blocks, each of which presents a series of words from two of three different affective categories: positive (for example, joyful), negative (for example, hopeless) and neutral (for example, element). The participant is given a target category and is asked to press the response pad only when they see a word matching this category. Some pairs of trials maintain the same target type (non-shift; easier), whereas others change the target type (shift; harder). Outcomes are category-specific reaction times, their differences and accuracy.

#### Facial emotion recognition task

This task assesses subjective perception of positive and negative social stimuli, i.e. facial expressions of six basic emotions, fear, anger, sadness, happiness, surprise and disgust. The version used here is adapted from Richards et al. (2002). The task is run on a computer using E-Prime (v.2, Psychology Software Tools, Inc., PA, USA) and presents participants with black and white images of a male face, front on, one face at a time (from the Ekman face set, Ekman and Friesen 1978). The expressions on the face are arrayed in blends of two emotions varying in ratios of 10 %:90 %, 30 %:70 %, 50 %:50 %, 70 %:30 % and 90 %:10 %. Participants are asked to rate each face for the intensity of one of the emotions, on a 9-point scale from 1 = ‘not at all (emotion)’ to 9 = ‘very (emotion)’. Each emotion is blended separately with two others in this way, thus providing an average rating of perceived emotional intensity over ten faces of varying blends. However, the rated response to the two 50 % blends for each emotion (e.g. fear with sadness and surprise) should provide the most reliable measure of perceptual bias, so these were analysed separately.

### Blood assays

Blood samples were centrifuged in Vacutainer™ lithium-heparin tubes at 4,000 rpm for 10 min at 4 °C. Then, 750 μl plasma supernatant was pipetted into each of two Eppendorf cups. These had been prepared with 120 μl 25 % (*w*/*v*) solution of sulfosalisylic acid, to deproteinise the plasma. The mixture was vortexed for 1 min until evenly milky, then frozen at −80 °C prior to transportation for assay. The amino acid assays were carried out by DSM Food Specialties B.V., Delft, NL. Plasma amino acid analysis was conducted with HPLC via a 2- to 3-mm Bischof Spherisorb ODS II column. The plasma TRP ratio was calculated by dividing the plasma TRP concentration by the sum of the other LNAA, i.e. valine, isoleucine, leucine, tyrosine and phenylalanine. All amino acid ratios are presented as molar weight ratios.

### Data analyses

The hypotheses were tested by comparing dependent variable means for significant differences between treatment groups on the treatment test day (Day 2), after adjusting for baseline performance (Day 1). This was achieved using analysis of covariance (ANCOVA) including Day 1 performance as a covariate. In addition, for the reading-dependent AGN test, errors on the NART (a measure of English verbal IQ) were included as a covariate. This variable did not differ by treatment group (see Table 2) but could still contribute to variance in performance because NART errors correlate positively with increased latency to respond and more omission errors in this task (e.g. Spearman’s rho = 0.30–0.50). These ANCOVAs also included planned contrasts between each LumiVida™ dose group and the control group (simple contrasts). Baseline means were compared by one-way ANOVAs or Kruskal–Wallis tests for non-parametric data. Where data were skewed greater than ±1, each variable was transformed appropriately, e.g. positive skew reduced by natural log (Ln) transformation, before analysis. Significance level (alpha) was taken as 0.05: where results were predicted by directional hypotheses, one-tailed probability (*p*) levels are reported; otherwise, two-tailed levels are given. Significance of multiple pairwise comparisons within a particular dependent variable was adjusted by Bonferroni correction or by use of Dunnett’s *t* test. Otherwise, alpha was not adjusted for numbers of tests of a priori hypotheses across multiple variables, as that would increase the risk of Type II errors, particularly for relatively small sample sizes.

**Table 2:** Age, NART errors, neuroticism and aggression by treatment group

Treatment group	Number	Age (years) mean (SD)	NART errors mean (SD)	Neuroticism mean (SD)	Aggression mean (SD)
Control	19	55.7 (6.1)	9.3 (4.7)	11.4 (8.5)	15.7 (6.3)
2 g LumiVida™	20	54.6 (5.5)	8.4 (5.3)	6.3* (4.5)	11.8* (2.7)
4 g LumiVida™	21	54.7 (6.9)	11.5 (6.1)	8.1 (5.5)	15.5 (6.1)

*p < 0.05, mean different from control; Dunnett’s *t* for multiple comparison to the control group

## Results

### Participant characteristics

Sixty women aged 45–65 years completed the study, 19 receiving the control treatment, 20 receiving 2 g LumiVida™ and 21 receiving 4 g LumiVida™. Their average age was 55.0 (SD = 6.1) years and as randomisation to group was stratified by age, treatment groups did not differ by age (Table 2, *F*(2, 57) < 1). The groups also did not differ significantly by NART errors (verbal IQ estimate) (Table 2, *F*(2, 57) = 1.82). There were also no differences in impulsivity or DASS-21 scores (depression, anxiety and stress) between groups (data not shown). In partial correlations controlling for body weight, these traits were not significantly related to baseline TRP/LNAA ratio, with the exception of impulsivity which was weakly negatively correlated (*r*(44) = −0.27, *p* < 0.05, one tail), in those participants able to give blood samples. However, despite double-blind randomisation, the 2-g LumiVida™ group was significantly lower in neuroticism (Dutch personality inventory) and total aggression (12-item aggression questionnaire) compared with the control group, *F*(2, 57) = 3.34 and 3.46, respectively, both *p* < 0.05 (Table 2). Thus, for mood and emotional processing tasks, these traits were examined for significant influence on the outcomes by ANCOVA.

Participants did not differ significantly in BMI across groups, with BMI ranging from 17.7 to 34.3 (mean (SD) = 24.9 (3.3)). Fifty-five per cent of participants had never smoked; the remainder being ex-smokers, although four participants admitted to occasionally having a ‘social’ cigarette.

### Plasma TRP and TRP/LNAA ratio

#### Blood assays

Given the collection difficulties, no blood was obtainable from 13 participants: three of the control, six of the 2-g LumiVida™ dose and four of the 4-g LumiVida™ dose. In addition, the second blood sample was not available for two participants from the 4-g LumiVida™ dose. Samples were assayed in duplicate: intra-assay coefficients of variation for TRP were 5.1 % for sample 1 (baseline) and 4.6 % for sample 2 (treatment test day), indicating highly reliable assay data. For three cases, results were only available from one sample (two cases for sample 1; one for sample 2). Distributions were normal.

#### Effects on plasma tryptophan

Plasma TRP pre- and posttreatment levels (samples 1 and 2) are given in Table 3. The baseline levels (sample 1) did not differ between treatment groups, one-way ANOVA group effect, *F*(2, 44) = 1.02, variance explained *η*_p_^2^ = 0.04, NS.

**Table 3:** Plasma TRP (μmol/g) for samples 1 (pretreatment) and 2 (posttreatment)

Treatment	Sample 1			Sample 2				
	Mean	SD	Number	Mean	SD	Number	Adj. mean	SD
Control	34.45	5.00	16	32.37	4.90	16	33.39	4.80
2 g LumiVida™	37.18	6.77	14	41.80	6.26	14	40.12	4.83
4 g LumiVida™	34.58	5.80	17	50.00	10.36	15	50.48	4.78
Total	35.31	5.86	47	41.18	10.43	45		

*Adj. mean* means adjusted for sample 1 levels

**Fig. 1:**
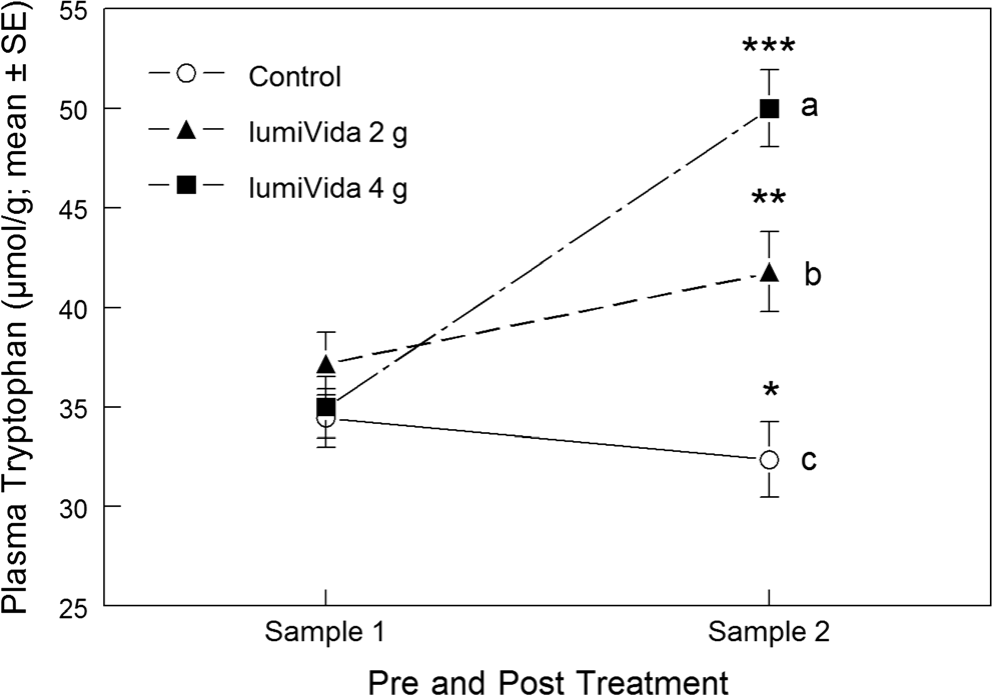
Effects of treatments on plasma TRP. *Different letters* indicate differences between treatment groups, adjusted for baseline levels. Within-group pre- vs. posttreatment comparisons: sample 2 differed from sample 1, *p < 0.02; **p < 0.01; ***p < 0.001

The impact of treatment condition on any change in plasma TRP was examined using one-way ANCOVA on the second TRP sample level, with sample 1 TRP levels as the covariate, i.e. testing for the group effect adjusted for any influence of baseline levels. Treatment had a highly significant effect on plasma TRP, ANCOVA group effect, *F*(2, 41) = 50.2, *p* < 0.001, *η*_p_^2^ = 0.71. Baseline (sample 1) TRP levels were a strongly significant covariate, *F*(1, 41) = 63.4, *p* < 0.001, *η*_p_^2^ = 0.61 (see Table 3). Compared with the control, TRP levels were significantly higher after both 2 and 4 g LumiVida™, and higher after 4 g than after 2 g, Bonferroni-adjusted pairwise comparisons, all *p* < 0.001 (Fig. 1).

Within each condition, sample 2 TRP levels were increased compared with sample 1 for both 2 g and 4 g treatments, paired *t*(13) = 3.52, *p* < 0.01, *t*(14) = 9.80, *p* < 0.001, respectively. Conversely, TRP levels for sample 2 were less than for sample 1, following the control treatment, paired *t*(15) = 2.86, *p* < 0.02 (Fig. 1). These differences between effects of treatments are supported by a significant Condition × sample interaction, RMANOVA *F*(2, 42) = 51.4, *p* < 0.001.

#### Effects on plasma TRP/LNAA ratio

Plasma TRP/LNAA pre- and posttreatment ratios (samples 1 and 2) are given in Table 4. The baseline ratio (sample 1) did not differ between treatment groups, one-way ANOVA group effect, *F*(2, 44) < 1, variance explained *η*_p_^2^ = 0.004, NS.

**Table 4:** Plasma TRP/LNAA for samples 1 (pretreatment) and 2 (posttreatment)

Treatment	Sample 1			Sample 2				
	Mean	SD	Number	Mean	SD	Number	Adj. mean	SD
Control	0.063	0.010	16	0.057	0.008	16	0.058	0.012
2 g LumiVida™	0.064	0.007	14	0.080	0.008	14	0.080	0.011
4 g LumiVida™	0.064	0.012	17	0.095	0.022	15	0.094	0.012
Total	0.064	0.010	47	0.077	0.022	45		

*Adj. mean* means adjusted for sample 1 ratios

**Fig. 2:**
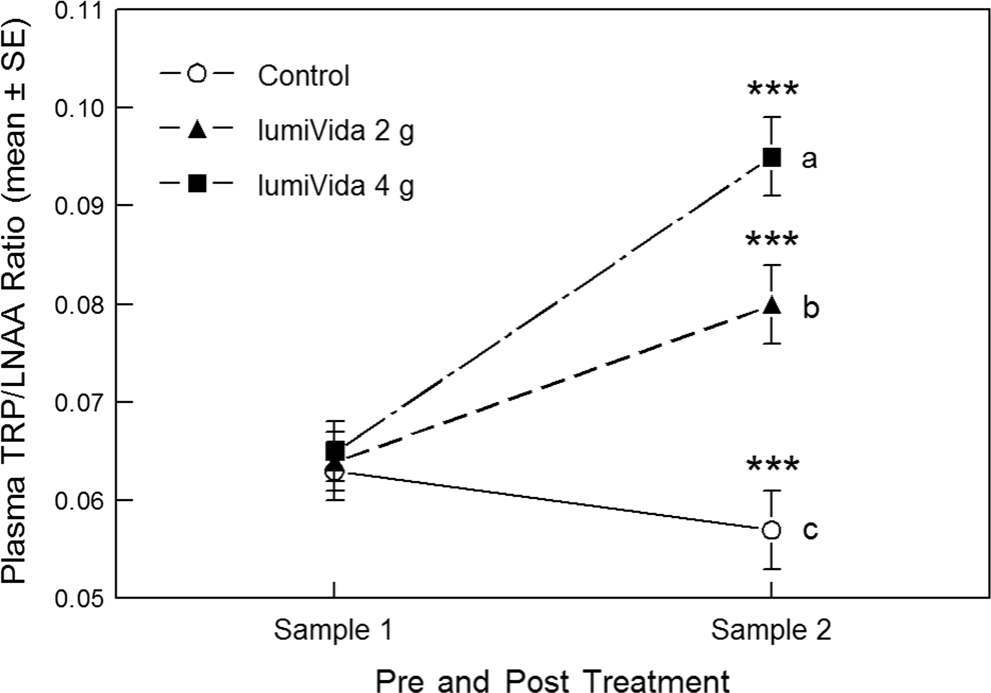
Effects of treatments on plasma TRP/LNAA ratios. *Different letters* indicate differences between treatment groups, adjusted for baseline ratios. Sample 2 differed from sample 1 within each treatment condition, ***p < 0.001

The impact of treatment condition on any change in plasma TRP/LNAA ratio was examined using one-way ANCOVA on the sample 2 (posttreatment) TRP/LNAA ratios, with sample 1 TRP/LNAA ratios as the covariate, i.e. testing for the group effect adjusted for any influence of baseline ratios. Treatment had a highly significant effect on plasma TRP/LNAA, ANCOVA group effect, *F*(2, 41) = 46.7, *p* < 0.001, *η*_p_^2^ = 0.70. Baseline (sample 1) TRP/LNAA ratios were a strongly significant covariate, *F*(1, 41) = 42.4, *p* < 0.001, *η*_p_^2^ = 0.51 (see Table 4). Compared with the control, TRP/LNAA ratios were higher after both 2 and 4 g LumiVida™, and higher after 4 g than after 2 g, Bonferroni-adjusted pairwise comparisons, control vs. 2 and 4 g, *p* < 0.001, 2 g vs. 4 g, *p* = 0.002 (Fig. 2).

Within each condition, sample 2 TRP/LNAA ratios were increased compared with sample 1 for both 2 and 4 g treatments, paired *t*(13) = 8.18, *p* < 0.001, *t*(14) = 7.58, *p* < 0.001, respectively: these increases in TRP/LNAA ratios corresponded to 25 and 48 % increases over baseline, respectively. Conversely, TRP/LNAA ratios for sample 2 were less than for sample 1 following the control intervention, paired *t*(15) = 4.44, *p* < 0.001, corresponding to a 9 % reduction from baseline (Fig. 2). These differences between effects of treatments are supported by a significant condition × sample interaction, RMANOVA *F*(2, 42) = 48.5, *p* < 0.001.

### Mental and physical sensations

#### Data reduction for mental and physical sensations

The recommended approach to analysing the 28 measures of MAPSS is first to reduce the number of measures to a smaller number of latent variables or factors using principal component analysis (PCA) (Rogers et al. 2010). The PCA was run on all measures of MAPSS, treating each time point as a separate case, in order to have enough cases (*n* = 240) relative to variables to make PCA reliable, and to allow testing for changes in the same latent variables over repeated sessions. Although this approach ignores the possibility that treatment conditions could contribute to within-subject variance across measurements, it is a common procedure in the literature, and using the alternative multilevel approach on our large variable to sample ratio presents additional problems of reliability (Reise et al. 2005). The initial solution, with varimax rotation, produced seven components with Eigen values of >1; however, observation of scree plot and factor loadings suggested that a five-factor solution would be a better fit, explaining 55 % of the variance in total.

The first two factors were the dominant components (Eigen = 7.5, 3.5; %variance = 13.9, 13.5, respectively). For each factor, combined variables were computed by averaging scores from all items that loaded >0.50, thus avoiding any items that had lower loadings on more than one factor. Thus, the first factor consisted of the following seven items, in order of loading: ‘relaxed/calm/at ease’, ‘able to concentrate/focus’, ‘mentally alert/attentive/observant’, ‘cheerful/happy/contented’, ‘clear-headed’, tranquil/peaceful’ and ‘energetic/active/strong/lively’. This factor was labelled ‘wellbeing’ (overall mean = 6.71).

The second factor consisted of six items, in order of loading: ‘muzzy/dazed/spaced out’, ‘fatigued/exhausted/worn out’, ‘strange/weird/not my usual self’, ‘sleepy/drowsy/half awake’, ‘headaches/feel headachy’ and ‘fidgety/twitchy’. This factor was labelled ‘fatigue’ (overall mean = 1.94).

The third factor consisted of four items (11.3 % of variance): ‘panicky/frantic’, ‘uneasy/apprehensive/concerned’, ‘fearful/scared/afraid’ and ‘anxious/worried/nervous’. This factor was labelled ‘anxiety’ (overall mean = 2.24).

The fourth factor consisted of six items (11.2 % of variance): ‘mind is racing’, heart is pounding/racing’, ‘buzzing/feel stimulated/hyper’, ‘agitated/restless/jumpy’, ‘impulsive/spontaneous’ and ‘tense/on edge’. This factor was provisionally labelled ‘agitation’ (overall mean = 1.32).

The fifth factor consisted of just two items (5.1 % of variance): ‘miserable/depressed/dejected’ and ‘hot/sweaty’. This factor was labelled ‘negative affect’ (overall mean = 1.34).

In addition, to check that this PCA result had not been substantially distorted by treatment effects, we also ran the PCA in the same way on the first baseline MAPSS measure only. Although this is not a reliable PCA given the large variable to sample ratio, nevertheless, the factor loading was very similar. The minor differences were that (a) two items, ‘fatigued’ and ‘headaches’, loaded weakly and negatively on the wellbeing factor (−0.50 and −0.57, respectively) rather than on the fatigue factor, while ‘strange’ did not load above 0.50 on any factor. (b) The agitation factor had a slightly higher Eigen value than the anxiety factor, but the structures were the same. Thus, it seems unlikely that treatment considerations in later mood measures had significantly distorted the factor loadings.

#### Effects on wellbeing

**Fig. 3:**
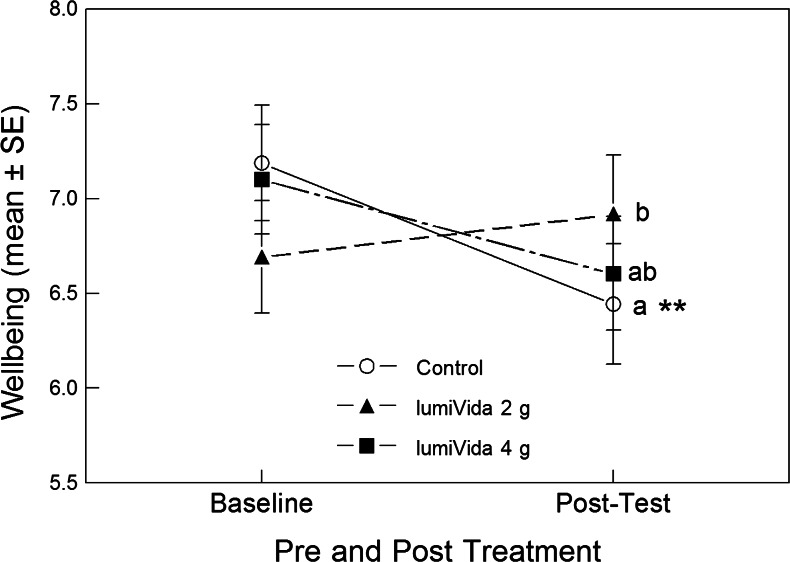
Effects of treatments on wellbeing at baseline and posttest. The significant pre–post by treatment interaction can be seen: 2 g LumiVida™ resisted any decline in wellbeing, and this effect was significantly different from control. The effect of 4 g was closer to control but did not differ significantly from either of the other groups. *Different letters* indicate differences between treatment groups, adjusted for baseline levels. Only in the control condition did the change (reduction) from baseline achieve significance (see below), **p < 0.01

Wellbeing raw scores were normally distributed and did not require transformation. Average wellbeing scores at baseline (before intervention drink on the treatment test day) and posttest are shown in Fig. 3. The baseline levels did not differ between treatment groups, one-way ANOVA group effect, *F*(2, 57) < 1, variance explained *η*_p_^2^ = 0.03, NS.

The impact of treatment condition on any change in wellbeing was examined using one-way ANCOVA on the posttest, and posttreatment, wellbeing level, with baseline levels as the covariate, i.e. testing for the group effect adjusted for any influence of baseline levels. Treatment condition had a significant though weak effect on wellbeing overall, ANCOVA group effect, *F*(2, 56) = 2.65, *p* < 0.05 one tail, *η*_p_^2^ = 0.09 (adjusted means (SD): control = 6.32 (1.10); 2 g = 7.11 (1.11); 4 g = 6.54 (1.10)). Baseline wellbeing levels were a strongly significant covariate, *F*(1, 56) = 33.02, *p* < 0.001, *η*_p_^2^ = 0.37. Compared with the control treatment, wellbeing levels remained significantly higher after 2 g (Bonferroni-adjusted pairwise comparisons, *p* < 0.05, one tail) but not after 4 g LumiVida™. The difference in wellbeing between the two dose groups was not significant. It can be seen in Fig. 3 that wellbeing declined from baseline to posttest in the control and (non-significantly) after 4 g LumiVida™ conditions but remained stable after 2 g LumiVida™.

Posttest change from baseline was tested for significance within each condition: posttest wellbeing levels were significantly lower than baseline after the control treatment, paired *t*(18) = 3.30, *p* < 0.01. However, neither the slight increase from baseline after 2 g (*t*(19) = 0.91) nor the decline after 4 g LumiVida™ (*t*(20) = 1.61) were statistically significant changes from baseline. These differences between effects of treatments on change from baseline are supported by a significant condition × pre–post-interaction, RMANOVA *F*(2, 57) = 3.53, *p* < 0.05, *η*_p_^2^ = 0.11. There was evidence for an average decline in wellbeing from baseline to posttest, pre–post-main effect, *F*(1, 57) = 4.78, *p* < 0.05, *η*_p_^2^ = 0.08 (see Fig. 3).

#### Effects on fatigue

Fatigue raw scores were positively skewed and so were Ln transformed. Average fatigue (transformed) scores at baseline and posttest are given in Table 5. The baseline levels did not differ between treatment groups, one-way ANOVA group effect, *F*(2, 57) < 1, variance explained *η*_p_^2^ = 0.02, NS.

**Table 5:** Effect of LumiVida™ on fatigue (Ln-transformed) for baseline (pretreatment) and posttesting on Day 2

Treatment	Baseline			Posttest				
	Mean	SD	Number	Mean	SD	Number	Adj. mean	SD
Control	0.51	0.51	19	0.91	0.68	19	0.85	0.48
2 g LumiVida™	0.36	0.40	20	0.36	0.42	20	0.40	0.47
4 g LumiVida™	0.40	0.36	21	0.59	0.53	21	0.61	0.47
Total	0.42	0.42	60	0.62	0.59	60		

*Adj. mean* means adjusted for baseline levels

**Fig. 4:**
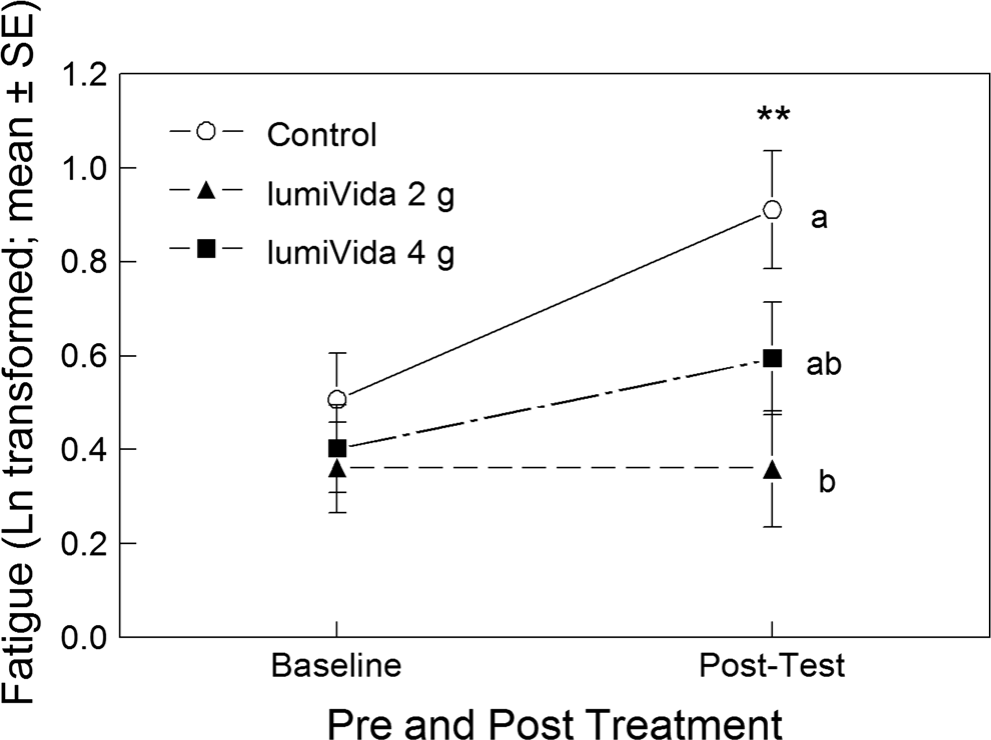
Effects of treatments on fatigue during testing. *Different letters* indicate differences between treatment groups, adjusted for baseline levels. The significant pre–post by treatment interaction can be seen (see text): 2 g LumiVida™ resisted any increase in fatigue, and this effect was significantly different from the control, whereas the increase was intermediate after 4 g. Posttest fatigue increased significantly from baseline only after the control treatment, **p < 0.01

The impact of treatment condition on any change in fatigue was examined using one-way ANCOVA on the posttest, and posttreatment, fatigue level, with baseline levels as the covariate, i.e. testing for group effect adjusted for any influence of baseline levels. Treatment condition had a significant effect on fatigue overall, ANCOVA group effect, *F*(2, 56) = 4.42, *p* < 0.01, one tail, *η*_p_^2^ = 0.14 (see adjusted means, Table 5). Baseline fatigue levels were a strongly significant covariate, *F*(1, 56) = 21.29, *p* < 0.001, *η*_p_^2^ = 0.28. Compared with the control treatment, fatigue levels remained low after 2 g LumiVida™, by the end of testing (simple contrast, *p* < 0.01, one tail). Fatigue after 4 g LumiVida™ was intermediate but not quite significantly different from the control (simple contrast, *p* = 0.06, one tail). It can be seen in Fig. 4 that fatigue increased from baseline to posttest in the control group and to a lesser extent after 4 g LumiVida™ but remained stable and low after 2 g LumiVida™.

Posttest change from baseline was tested for significance within each condition: posttest fatigue levels were significantly greater than baseline after the control treatment, paired *t*(18) = 3.13, *p* < 0.01. However, the slight increase from baseline after 4 g LumiVida™ was not significant (*t*(20) = 1.81, *p* = 0.09), and fatigue did not increase after 2 g LumiVida™, *t*(19) = 0.04, NS. These differences between effects of treatments on change from baseline were supported by a significant condition × pre–post-interaction, RMANOVA *F*(2, 57) = 3.39, *p* < 0.05, *η*_p_^2^ = 0.11. There was evidence for an overall increase in fatigue from baseline to posttest, pre–post-main effect, *F*(1, 57) = 9.76, *p* < 0.01, *η*_p_^2^ = 0.15 (see Fig. 4).

### Affective go/no go task

#### Specific data analyses issues

Dependent variables in this task include: latency to respond to the target word; commission errors, when the key is wrongly pressed to a distractor word; omission errors, when the key is *not* pressed to a target word. Although the task outcomes can be broken down into sub-trials varying in distractor valence, for example, for the purposes of this report, the analyses are of target valence only (positive or negative), i.e. neutral distractor trials are combined with either positive or negative distractor trials, depending on the target. One participant was unable to complete the AGN task on Day 2, so she was excluded from these analyses.

#### Effects on response latencies

**Fig. 5:**
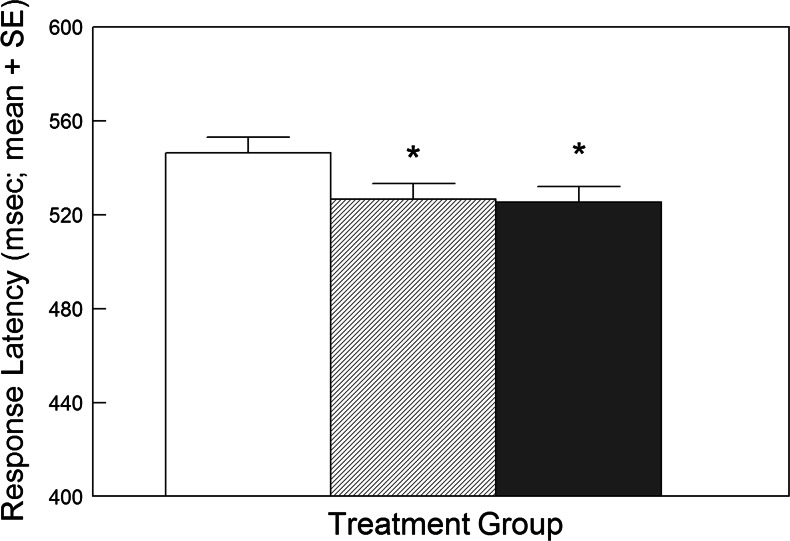
Effect of LumiVida™ on latencies to respond to positive target words (with negative or neutral distractor words; all trials). Both doses of LumiVida™ (hashed column = 2 g; solid column = 4 g) reduced the response latency, *p < 0.05 vs. control (open column). Means are adjusted for baseline performance, age and NART errors

It was predicted that participants would respond faster to positive words with negative or neutral distractors after LumiVida™ if the treatment reduced bias to negative stimuli. For both doses, LumiVida™ reduced the latency to respond to positive words, i.e. faster reactions, ANCOVA group effect, *F*(2, 53) = 3.05, *p* < 0.03 one tail, *η*_p_^2^ = 0.10; contrasts, 2 g vs. control, *p* < 0.05, 4 g vs. control, *p* < 0.05 (Fig. 5). This suggests lessening of interference from negative or neutral distractor words by the active treatment. Neither neuroticism nor aggression scores were significant covariates.

Response latencies for negative target words were unaffected by treatment condition (data not shown).

#### Effects on errors of omission

**Fig. 6:**
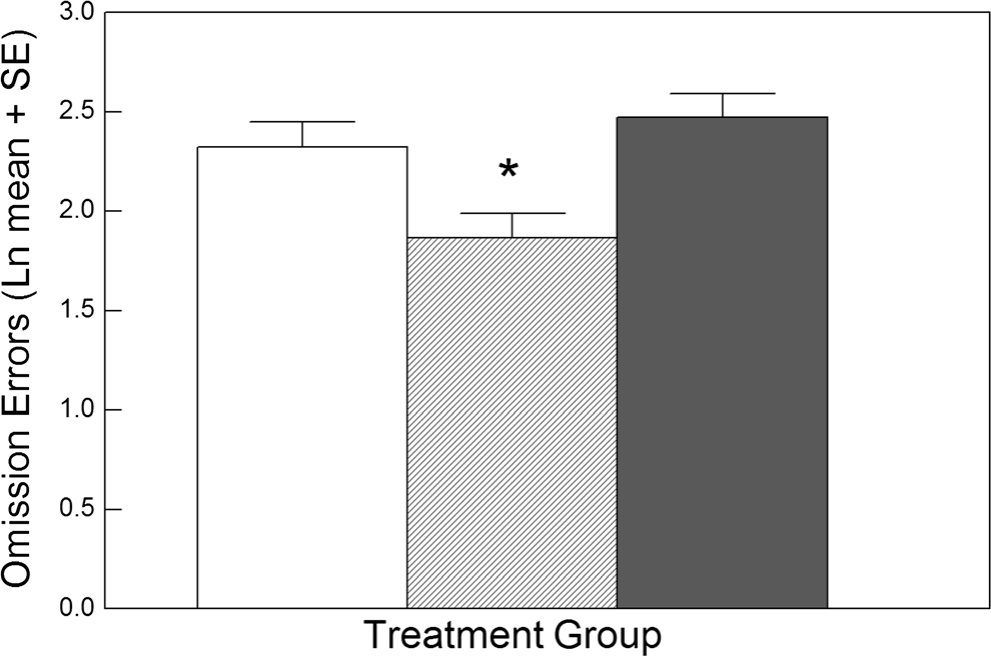
Effect of LumiVida™ on errors of omission for positive target words (with negative or neutral distractor words; all trials). The lowest dose (2 g) of LumiVida™ (hashed column) significantly reduced the number of errors, *p < 0.02 vs. control (open column); 4 g LumiVida™ (solid column) did not differ from the control. Means are adjusted for baseline performance, age, NART errors and neuroticism. Data are natural log transformed to normalise positive skew

These errors indicate failing to press the key in response to a target word. It was predicted that LumiVida™ would reduce such errors when the target was positive and distractors were negative or neutral. Similarly to latency data, there was a significant effect of treatment for the positive target condition; moreover, neuroticism was a significant covariate for this variable, *F*(1, 52) = 6.10, *p* < 0.02, *η*_p_^2^ = 0.11, and the resulting adjusted main effect of treatment was strongly significant, ANCOVA group effect, *F*(2, 52) = 6.49, *p* < 0.01, *η*_p_^2^ = 0.20. Planned contrasts showed that this effect was due to a reduction in omissions after 2 g LumiVida™ vs. control, *p* < 0.02, but not after 4 g LumiVida™ vs. control, *p* > 0.1 (Fig. 6). This suggests that the presence of negative or neutral distractors produced less interference in accuracy of responding to positive targets after the low dose of LumiVida™. There were no significant effects of treatment on omission errors for negative target words (*F* < 1.62). There were also no effects of treatment on errors of commission (i.e. incorrect key pressing to a distractor word; *F* < 1).

### Facial emotional expression rating task

#### Specific approaches to data analysis

**Fig. 7:**
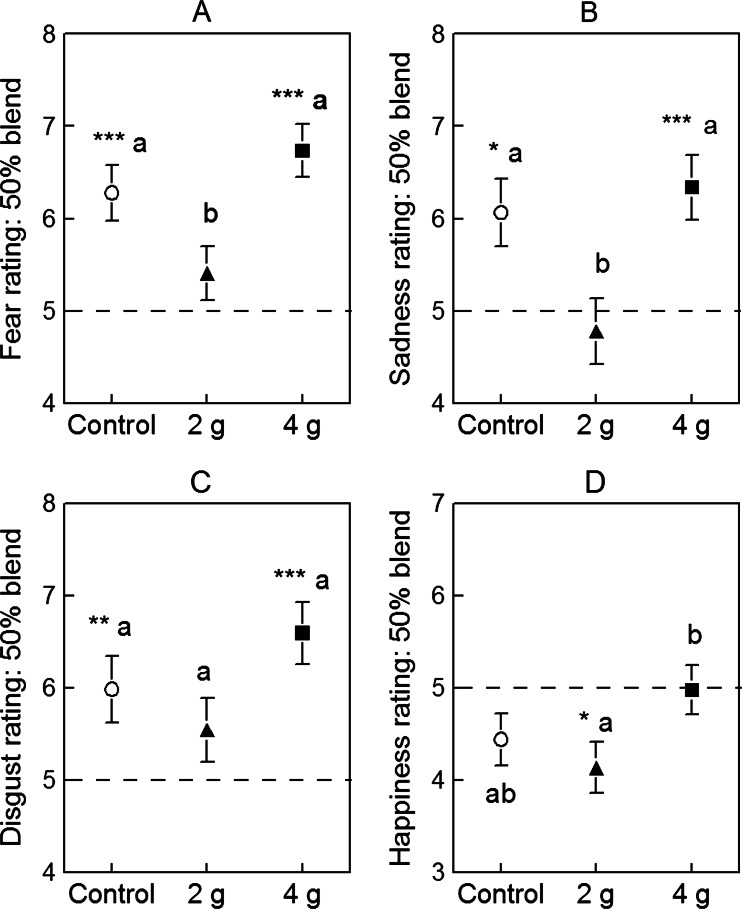
Effects of LumiVida™ treatments on perception of particular emotions in faces with 50 % blends of those emotions: **a** fear, **b** sadness, **c** disgust, and **d** happiness. The mid-point of 5 (*broken line*) on the 1–9 scale represents accurate perception of 50 % of the expression present (by comparison with the other % blends). Data are expressed as mean ± SE for Day 2 ratings, adjusted for baseline (Day 1). Significant differences from mid-point rating are indicated (one-sample *t* tests), *p < 0.05; **p < 0.01; ***p < 0.001. *Differing letters* (a and b) indicate significant differences between groups

Following the ANCOVA including baseline day (Day 1) performance as a covariate, comparisons between the control and LumiVida™ groups were made using planned simple effect contrasts. Post hoc comparisons between means for the two doses of LumiVida™ were Bonferroni adjusted. These analyses were performed on the overall average rating including all ten blends of a given emotion, and separately on ratings for faces with 50 % of a given emotion (averaged over trials against two other emotions). In addition, for the 50 % blend data, one-sample *t* tests were used to test for mean differences from the scale mid-point of 5, as a measure of accuracy of emotional perception (significances indicated in Fig. 7 and its legend). For this test, subjective ratings of emotional intensities are the dependent variables, so age and NART errors are not included as covariates; these latter variables did not differ by treatment. Neuroticism and aggression scores were tested for significance of covarying: aggression was never a significant covariate; neuroticism was significant for anger and happiness, as described below. The dependent variables and covariates reported here were not skewed, so raw scores were analysed.

#### Effects on fear

Treatment condition significantly altered rated intensity of expression of fear in 50 % blended faces, one-way ANCOVA, *F*(2, 56) = 5.40, *p* < 0.01, *η*_p_^2^ = 0.162: rated fear was less after 2 g LumiVida™ vs. control (*p* < 0.05), whereas ratings did not differ between 4 g LumiVida™ and control (Fig. 7a). The difference between LumiVida™ doses was significant, *p* < 0.01.

The results for the overall average fear rating were similar (Table 6, there was a significant effect of treatment condition, *F*(2, 56) = 3.38, *p* < 0.05, *η*_p_^2^ = 0.108, with less rated fear after 2 g LumiVida™ than after the control, although this effect was not quite significant (*p* = 0.055, one tail). Fear rating after this dose was significantly less than after 4 g LumiVida™ (*p* < 0.05), which did not differ from the control.

**Table 6:** Effect of LumiVida™ on total ratings of emotional expressions: data are means (SE) over ten blends

Treatment group	Number	Fear	Sadness	Anger	Disgust	Surprise	Happiness
Control	19	6.00a (0.20)	5.14 (0.22)	5.57 (0.16)	5.59 (0.25)	6.26 (0.16)	5.00 (0.16)
2 g LumiVida	20	5.55**b** (0.19)	5.19 (0.22)	5.56 (0.16)	5.39 (0.24)	5.92 (0.15)	4.84 (0.16)
4 g LumiVida	21	6.24a (0.19)	5.61 (0.21)	5.54 (0.15)	5.79(0.23)	5.99 (0.15)	5.21 (0.16)

Means adjusted for baseline. Differing lowercase letters (a and **b**) indicate differences between means for fear ratings; overall fear rating after 2 g LumiVida™ was less than those for control (*p* < 0.06) and 4 g LumiVida™ (*p* < 0.05). No other emotion means differed on this measure

#### Effects on sadness

As with fear, treatment condition significantly altered rated intensity of sadness in 50 % blended faces, one-way ANCOVA, *F*(2, 56) = 5.44, *p* < 0.01, *η*_p_^2^ = 0.163: rated sadness was less after 2 g LumiVida™ vs. control (*p* < 0.05), whereas ratings did not differ between 4 g LumiVida™ and the control (Fig. 7b). The difference between LumiVida™ doses was significant, *p* < 0.01. For the overall average sadness rating, there was no effect of treatment condition, *F*(2, 56) = 1.45, NS, *η*_p_^2^ = 0.049, and no difference between doses.

#### Effects on anger and surprise

There was no effect of treatment on anger ratings for 50 % blended faces (*F* < 1, *η*_p_^2^ = 0.022), nor on overall anger ratings (*F* < 1, *η*_p_^2^ = 0.00). Including neuroticism as a significant covariate (*F*(1, 55) = 5.89, *p* < 0.02) for the 50 % blended faces ratings did not alter the results.

There was no effect of treatment on surprise ratings for 50 % blended faces, (*F* < 1, NS, *η*_p_^2^ = 0.005), nor on overall surprise ratings, (*F*(2, 56) = 1.27, NS, *η*_p_^2^ = 0.043).

#### Effects on disgust

There was a weak effect of treatment condition on ratings of disgust for 50 % blends, *F*(2, 56) = 2.43, *p* < 0.05 one tail, *η*_p_^2^ = 0.080, with a slightly lower average rating after 2 g LumiVida™ and higher rating after 4 g, although neither group differed significantly from the control (Fig. 7c). There was a trend for disgust to be rated lower after 2 g than after 4 g LumiVida™ (*p* < 0.10). For the overall average disgust rating, there was no effect of treatment condition (*F* < 1, *η*_p_^2^ = 0.026) and no difference between doses.

#### Effects on happiness

There was a significant effect of treatment on ratings of happiness for 50 % blends, when adjusted for the effect of neuroticism as a significant covariate (*F*(1, 55) = 4.97, *p* < 0.05, *η*_p_^2^ = 0.08), treatment effect, *F*(2, 55) = 3.22, *p* < 0.05, two tail, *η*_p_^2^ = 0.11. There were no differences between the control and either 2 or 4 g LumiVida™ but a higher rated perception of happiness after 4 g compared with 2 g LumiVida™ (Fig. 7d: *p* < 0.05, two tail, Bonferroni corrected).

For the overall average happiness ratings, there was no effect of treatment condition, *F*(2, 55) = 2.11, NS, *η*_p_^2^ = 0.07), and no difference between doses, when covarying for a significant effect of neuroticism (*F*(1, 55) = 5.03, *p* < 0.05, *η*_p_^2^ = 0.08), or when not.

## Discussion

### Effects of treatment on plasma TRP and TRP/LNAA

The expected dose-dependent increases in plasma TRP and TRP/LNAA ratio were seen after active treatments: both 2 and 4 g LumiVida™ were effective in raising plasma levels of TRP and the TRP/LNAA ratio, 90 min after administration. The mean changes were 25 and 48 % above baseline, respectively. These changes compare well with levels achieved by studies using TRP-rich α-lactalbumin (Booij et al. 2006; Merens et al. 2005), and also with pharmacokinetic data for 4 g LumiVida™ (Mitchell et al. 2011), and would be expected to raise synthesis of serotonin in the brain; however, in a previous report concerning LumiVida™, TRP levels and mood, the dose that produced a substantial and lasting improvement in mood was at least 5-fold greater (Markus et al. 2008). It is also worth noting that the absolute doses of TRP (0.08–0.16 g) are considerably smaller than typically used in studies assessing effects of TRP alone (e.g. 0.5–7 g). Moreover, given that equivalent increases in TRP/LNAA after α-lactalbumin are seen at doses containing about 0.5 g TRP, this suggests that the LumiVida™ peptide preparation is substantially more efficient in raising TRP/LNAA than α-lactalbumin; the TRP/LNAA content ratio is at least double that in α-lactalbumin, though it should be considered that LumiVida™ might also provoke a greater insulin response, thereby increasing peripheral uptake of competing LNAAs, particularly the branched chain amino acids.

The control drink, containing 3.11 g casein hydrolysate, slightly, but significantly, reduced TRP/LNAA relative to baseline (9 %): this is to be expected given the increase in LNAA that would result from casein hydrolysate intake, although there was no fasting comparison group in this study to rule out a decline due to circadian changes.

### Mental and physical sensations

Effects on mood, arousal and other physical sensations were assessed using ratings made on a computer (Rogers et al. 2010), at baseline and after performance testing. Treatment effects were found for the two dominant components, labelled wellbeing and fatigue, following principal components analysis.

Wellbeing tended to decline by the end of the testing period. This decline was prevented specifically by 2 g LumiVida™, though not by 4 g. One explanation for this ‘inverted-U’ dose–response effect could be that the higher dose produced other effects that began to counteract any improvement in wellbeing. In this respect, it is therefore interesting that the effect on fatigue was also strongest for the lower dose: 2 g LumiVida™ prevented any increase in fatigue after testing, which was clearly apparent for the control group, whereas 4 g had an intermediate effect. One study using α-lactalbumin to raise TRP availability to the brain reported increased nausea at a dose that produced less than double the TRP/LNAA ratio to that seen here (Scrutton et al. 2007). Inverted-U-shaped responses have been reported in the literature after TRP supplementation: α-lactalbumin supplementation trials that increased plasma TRP/LNAA to 21–67 % did not have an effect on mood (Booij et al. 2006; Markus et al. 1998, 2000, 2008; Merens et al. 2005; Scrutton et al. 2007). In a recent study comparing a higher dose of egg-white protein hydrolysate (containing 0.8 g TRP) against casein (providing a 10-fold difference in TRP/LNAA content—not plasma—ratio), the egg-white protein hydrolysate induced an increase in feelings of vigour, whereas this positive mood state declined after casein (Firk and Markus 2009). Conversely, a 15-fold increase in TRP/LNAA achieved after an intravenous injection of 7 g pure TRP was shown to impair mood and cognition (Sobczak et al. 2002a, 2003). Thus, there may be an inverted U-shaped relationship between TRP/LNAA ratios and mood in healthy subjects.

Nevertheless, adverse effects of TRP loading are more usually seen at much higher doses (Silber and Schmitt 2010), and there is a previous report that an acute 12 g LumiVida™ dose improves mood (Markus et al. 2008). Even so, it should be noted that, in that study, participants were not engaged in stressful performance testing, which itself can affect mood as well as potentially the serotonin system (Gibson and Green 2002; Robinson et al. 2010); indeed, stress is known to upregulate the enzymes TRP pyrrolase and indoleamine-2,3-dioxygenase involved in catabolysing TRP, and thus potentially reducing its availability for brain serotonin synthesis (Russo et al. 2003).

Anxiety was relatively low, though levels were slightly higher before than after testing: treatment had no effect on anxiety. The remaining mood states (agitation, negative affect) were at low levels and were not affected by treatment.

These findings are most appropriately compared with previous research using healthy participants, as opposed to those with a (family) history of affect disorders. Firstly, it is interesting to note that the beneficial effects of 2 g LumiVida™ on wellbeing are echoed by that dose’s prevention of fatigue caused by the testing. As the wellbeing measure included terms related to arousal and alertness, both mood effects might reflect a slight underlying stimulant effect of 2 g LumiVida™. Although some other studies have reported positive effects on mood after TRP manipulation, these often involved substantially higher doses of TRP and thus greater changes in TRP/LNAA (Charney et al. 1982). Moreover, it is not clear to what extent those improvements might reflect anxiolytic and even sedative effects, which are often observed at higher TRP doses (Fernstrom 2012; Leathwood and Pollet 1982; Sobczak et al. 2003). There are also several studies that have failed to find reliable mood changes by TRP supplementation (Merens et al. 2005; Scrutton et al. 2007), though others have demonstrated benefits to mood interacting with social behaviour context (Young 2013). Observed effects may depend on the variety of mood measures that have been used; the MAPSS measure used here might be particularly sensitive in the context of this study, but there are no previous reports of effects of TRP or 5-HT changes on this measure.

### Emotional processing and sensitivity

There is an expanding literature examining effects of serotonin manipulations on aspects of emotional processing; the interest arises from attempts to understand the role of serotonin in affect disorders such as depression and anxiety (Cowen 2008; Robinson et al. 2010; Robinson and Sahakian 2009). However, there are fewer studies that have looked at TRP supplementation on such effects in healthy participants. The theory is that in states of low serotonin activity, such as in depression, there is an attentional bias toward negative stimuli (Harmer 2008; Mathews and MacLeod 2005). Therefore, emotional processing is assessed by comparing responses to negative vs. positive stimuli; for example, speed and accuracy of recognition of pleasant and unpleasant emotional expressions or responses to target vs. distractor emotional words (Mathews and MacLeod 2005).

However, the results have not been entirely consistent; one oral dose of 1.8 g TRP enhanced recognition of both happy and fearful faces (Attenburrow et al. 2003), whereas 3 g/day TRP for 14 days increased recognition of happiness but decreased recognition of disgust expressions, and reduced attention to negative stimuli, suggesting a shift in bias toward positive stimuli (Murphy et al. 2006). Such effects are more often seen in women than men (Murphy et al. 2006).

In this study, LumiVida™ produced faster and more accurate (fewer omissions) responding to positive target words, which might reflect less distraction by, and thus a shift in attention from, negative words, as well as increased attention to positive words. It is notable that the most reliable effect was again for the 2 g dose—only this dose improved accuracy, although both doses were equally effective in reducing the latency to respond. Moreover, in the test of perception of emotional expressions, 2 g was again the effective dose, producing reductions in, and increased accuracy of, ratings of fear and sadness: one exception was a slightly more accurate perception of 50 % happy expressions after 4 vs. 2 g LumiVida™. These findings are consistent with LumiVida™ increasing serotoninergic activity in pathways processing emotional stimuli, leading to a potential anti-depressant like activity of a shift in emotional processing away from negative bias (Bari et al. 2010). Importantly, we controlled for any influence of neuroticism here, as such a ‘threat sensitivity’ aspect of personality has been shown to interact with serotoninergic modulation of brain processing of emotional expressions (Cools et al. 2005). It is worth emphasising that, although the group receiving the effective 2 g dose was also the least neurotic and aggressive of the three groups, these personality differences were adjusted for by analysis of covariance, and either had no impact, or, if anything, strengthened the effect of that dose. This argues against personality differences contributing to the lack of linear dose-response results, but given the relevance of these traits to serotonin function, the possibility of residual confounding must be acknowledged.

## Summary

Consistent with accumulating evidence from both enhancement and depletion of serotonin function (Murphy et al. 2006; Robinson and Sahakian 2009; Silber and Schmitt 2010), the most reliable effects of LumiVida™ across the tasks have been on processing of emotionally relevant stimuli. The direction of these effects is in line with the widely acknowledged antidepressant function of enhanced serotonin neurotransmission, i.e. a shift in processing bias from negative towards positive stimuli (Bari et al. 2010; Sharp and Cowen 2011).

One limitation of this study was restriction of participants to middle-aged women, who may be more susceptible to dietary TRP manipulation than men (Murphy et al. 2006; Silber and Schmitt 2010). Moreover, there was no fasted-only condition, i.e. all comparisons are made to a casein hydrolysate control treatment, which, while matching for energy and protein intake, may not be inert itself on the behaviours considered here. Nevertheless, it is possible that comparison with a fasted group may have exaggerated beneficial effects of LumiVida™. Still, the lack of linear dose-response results in this study questions whether the mechanism of action is the hypothesised increased release of serotonin per se. The pattern of results could depend on individual differences in susceptibility of brain serotonin function to dietary manipulation, although this was not indicated by the personality measures examined here. A possible role for serotonin transporter receptor gene polymorphisms remains to be explored in our data.

In conclusion, firstly our data provide the evidence that consumption of LumiVida™ does not induce any negative effects. Furthermore, these findings encourage the conclusion that daily consumption of a low dose of LumiVida™ may have beneficial effects on emotional function that may promote feelings of wellbeing; however, further work with such chronic dosing is required to be confident of this. These acute results suggest the potential for some emotional benefits, particularly during demanding occasions, and thus could confer resistance to depressive episodes.
